# Influence of Cu Content on the Microstructure and Mechanical Properties of Cr-Cu-N Coatings

**DOI:** 10.1155/2018/6491279

**Published:** 2018-01-14

**Authors:** Ji Cheng Ding, Teng Fei Zhang, Zhi Xin Wan, Hai Juan Mei, Myung Chang Kang, Qi Min Wang, Kwang Ho Kim

**Affiliations:** ^1^School of Convergence Science, Pusan National University, Busan 609-735, Republic of Korea; ^2^Global Frontier R&D Center for Hybrid Interface Materials, Pusan National University, Busan 609-735, Republic of Korea; ^3^The Institute of Materials Technology, Pusan National University, Busan 609-735, Republic of Korea; ^4^School of Electromechanical Engineering, Guangdong University of Technology, Guangzhou 510006, China

## Abstract

The Cr-Cu-N coatings with various Cu contents (0–25.18 (±0.17) at.%) were deposited on Si wafer and stainless steel (SUS 304) substrates in reactive Ar+N_2_ gas mixture by a hybrid coating system combining pulsed DC and RF magnetron sputtering techniques. The influence of Cu content on the coating composition, microstructure, and mechanical properties was investigated. The microstructure of the coatings was significantly altered by the introduction of Cu. The deposited coatings exhibit solid solution structure with different compositions in all of the samples. Addition of Cu is intensively favored for preferred orientation growth along (200) direction by restricting in (111) direction. With increasing Cu content, the surface and cross-sectional morphology of coatings were changed from triangle cone-shaped, columnar feature to broccoli-like and compact glassy microstructure, respectively. The mechanical properties including the residual stress, nanohardness, and toughness of the coatings were explored on the basis of Cu content. The highest hardness was obtained at the Cu content of 1.49 (±0.10) at.%.

## 1. Introduction

Transition metal nitride coatings, which possess superior properties such as high hardness, excellent thermal stability, good chemical inertness, and high wear resistance, have been intensively studied in many groups [1–3]. Chromium nitride (CrN), as one member of the transition metal nitrides, is an important hard coating material which is widely used in industrial application for improving life and working efficiency of cutting tools due to its relatively high hardness, superior resistance to high temperature oxidation, and corrosion resistance [4–7]. Toughness is an important factor for hard coatings applied in mechanical applications. Without improvement of toughness, the life time of CrN coatings can be dramatically reduced because of the propagation of cracks and abrasive wear at harsh working conditions. In order to achieve long-life time and high stability, several researches have been actively engaged in improving both the hardness and toughness of the nitride hard coatings, simultaneously. Incorporating a third soft metal phase into CrN based coatings is an easy way to satisfy above demands [8–12], which can adjust, for example, the microstructure, hardness, and surface quality of the coatings. Wu et al. [13] have deposited Cr-Nb-N coatings by magnetron sputtering in an Ar-N_2_ atmosphere and suggested that the hardness and oxidation resistance could be improved by addition of niobium. Kim et al. [14] have reported the effect of soft metal Ni-doping on the microstructure and mechanical properties, especially, on the toughness of the Cr-Ni-N coatings. Musil et al. [15] selected Cu as the third soft metal to be added to CrN coating system, and the result showed that the hardness up to 35 GPa was achieved at Cu content value of 1 at.%. Tan et al. [16, 17] synthesized Cr-Cu-N coatings by RF/DC reactive magnetron sputtering and investigated microstructure and hardness of the coatings by Cu addition where hardness reached over 27 GPa at low Cu content. Such a high hardness may be the consequence of the combination of grain refinement hardening and blocking of grain boundary sliding. Lee et al. [18] deposited the Cr-Cu-N coatings with Cu contents ranging from 0.4 to 14.9 at.%. The maximum average hardness around 20 GPa and scratching coefficient around 0.1 were found in the coatings with around 2.1 to 2.6 at.% Cu.

Previous works mainly focused on the influence of Cu doping on the microstructure and hardness, but the chemical status of Cu in the coatings, the tribological properties, and toughness of the coatings can be a topic of further investigations. Therefore, in this work, a more systematic study has been performed to identify the effect of various Cu contents on the phase structure, microstructure, and mechanical, tribological, and toughness performances of the Cr-Cu-N coatings.

## 2. Experiment Details

Six series of Cr-Cu-N coatings with various Cu content were deposited by hybrid coating system combining a radio frequency (RF) and a pulsed DC (PDC) sputtering, which was schematically shown in previous reports [19]. The Cr target and Cu target were connected with PDC and RF power sources, respectively. Various contents of Cu were obtained by adjusting the RF power of Cu target. In order to guarantee the homogeneous feature along the surface plane, the substrate holder was located at the center of vacuum chamber and then rotated continuously at 10 r/min during the deposition process. The distance between the samples and targets was approximately 100 mm. A bias power source was connected to the substrate holder. SUS 304 stainless steel materials and Si wafers were used as substrates. This stainless steel has the following chemical composition (in wt%): C (0.044), Si (0.43), Mn (1.12), P (0.032), S (0.004), Ni (8.03), Cr (18.13), N (0.04), and Fe in balance. Before actual experiment, all the substrates were ultrasonically cleaned in acetone and industrial alcohol sequentially for 30 min and then fixed on the holder after being blown dry with pure nitrogen. The chamber was evacuated to a base pressure below 6.0 × 10^−3^ Pa using rotary and turbomolecular pumps. Before deposition, ion bombardment was conducted by Ar glow discharge for 5 min with a bias voltage of −800 V at 0.8 Pa, and then plasma etching was conducted for 10 min with PDC sputtering (0.8 kW) and bias voltage of −800 V. To improve the adhesion of coatings, Cr interlayer was sputtering for 10 min. The working pressure was maintained to 0.4 Pa and the reactive gas N_2_ was constant at 20 sccm. To maintain the deposition pressure, the Ar gas flow rate was varied from 65 to 80 sccm. The duty cycle of the PDC power and the frequency of RF were maintained at 60% and 13.8 kHz, respectively. The Cr target power was kept at 0.8 kW, while the Cu target power was varied from 0 to 150 W to study the Cu content effect on the microstructure and mechanical properties of produced samples. The deposition time was 120 min and the deposition temperature was maintained at 300°C. The DC bias voltage was −150 V. The deposition parameters are listed in Table 1. During the whole deposition processes, no apparent micro-arcs were observed.

Chemical elemental proportions, over coating surfaces, were evaluated using electron probe micro analysis (EPMA, CAMECA SX100). The physical structures of various coating were determined by an X-ray diffractometer (XRD, D8-Discovery Brucker, Cu K*α*, 40 kV, 40 mA) patterns obtained from 20° to 80°. The chemical bonding status in coatings was evaluated by X-ray photoelectron spectra (XPS, VG Scientifics, ESCALAB 250), using the Ar^+^ ions etching sample surface for 300 s, and the spectra were calibrated for the value of carbon peak C 1s at 284.6 eV. The morphologies of the Cr-Cu-N coatings were measured by using field-emission scanning electron microscopy (FE-SEM, S4800, Hitachi). The coatings thickness was measured from the cross-sectional images and deposition rate was calculated through the individual deposition time. The hardness and Young's modulus of the coatings were confirmed by using nanoindentation tester (Hysitron, TI950 TriboIndentor) under a load of 6 mN. About twenty-five indentations were performed for each sample and an average value was proposed. The surface average roughness of the coating was measured over a scan area of 5 × 5 *μ*m^2^ using an atomic force microscope (AFM, MFM-3D, Asylum Research) in contact mode. Residual stress was calculated from the stress tester machine (J&L Advanced Plasma Technology™) according to substrate curvature method based on the Stoney equation [20]. The friction coefficients were measured through sliding wear test using a conventional ball-on-disk wear apparatus. An alumina ball with 6 mm diameter was used as a counterpart material. The sliding tests were conducted with a linear speed of 100 mm/s under a load of 2 N at ambient temperature around 24°C and a relative humidity of 37%. The sliding distance was 60 m in each sample. Finally, the toughness of the coatings was evaluated by using Vickers hardness testing system (Mitutoyo HM-220) at 2 N load.

## 3. Results and Discussion

### 3.1. Studies on Chemical Composition and Deposition Rate

**Figure 1:**
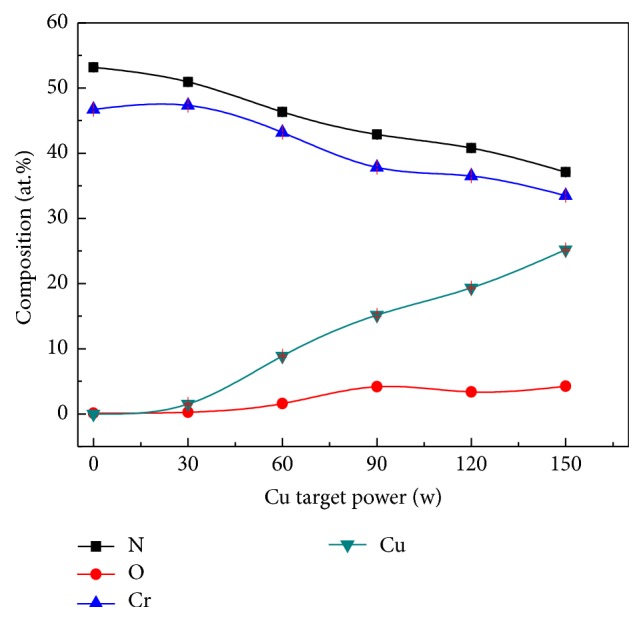
The composition of the Cr-Cu-N coatings with respect to the Cu target power.

**Figure 2:**
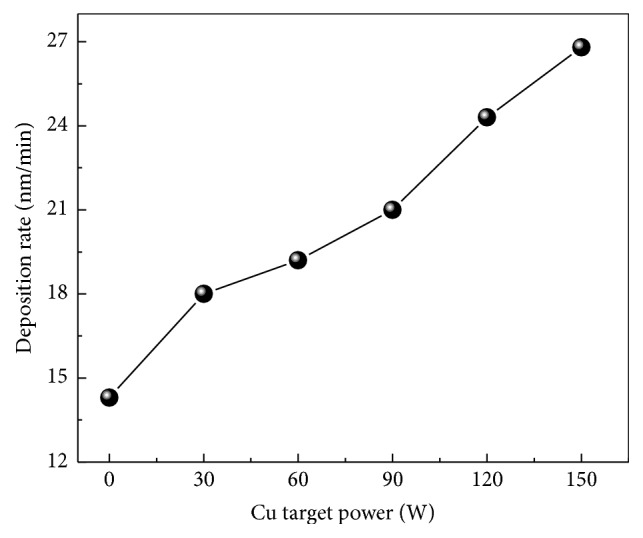
The relation between deposition rate and Cu target power.

Figure 1 shows the chemical composition analysis of the coatings measured by EPMA. The concentration of oxygen inside the coatings was less than 5 at.% from all the deposited samples, which may be attributed to residual gas in the chamber during deposition, and the coating was close to stoichiometric in pure CrN. The Cu content presents a nearly linear increase from 0 at.% to 25.18 (±0.17)  at.% with increasing the Cu target power, while the N and Cr contents decrease monotonically from 53.19 (±0.19)  at.% and 46.71 (±0.26) at.% to 37.10 (±0.15)  at.% and 33.49 (±0.21)  at.%, respectively. It is noted that, with increasing Cu content, the coatings become N deficient and at the maximum Cu content of 25.18 (±0.17)  at.%, the atomic ratio of metal versus nitrogen increased to about 1.6 : 1. That is attributed to the atomic mass of N atoms that is lighter than Cr atoms; thus, N atoms are prone to being sputtered by impinging ions with high energy [21, 22].  Figure 2 shows the variation of deposition rate together with the changed Cu target sputtering power. It is clearly seen that the deposition rate of coatings increased from 14.3 to 26.8 nm/min as the sputtering power increased from 0 to 150 W, which was attributed to more ionization ions that could reach effectively to the substrate surface. Le et al. [23] reported the effect of sputtering power on the growth rate of films where they believed that, at higher sputtering power in magnetron sputtering system, adatoms acquire sufficient kinetic energy from the high energy inert argon gas. Then the surface diffusion of these adatoms is expected to improve with the momentum which in turn transfers to the nucleation and growth of films; thereby the films thickness was increased with sputtering power.

### 3.2. The Phase Structure and Microstructure Analyses

**Figure 3:**
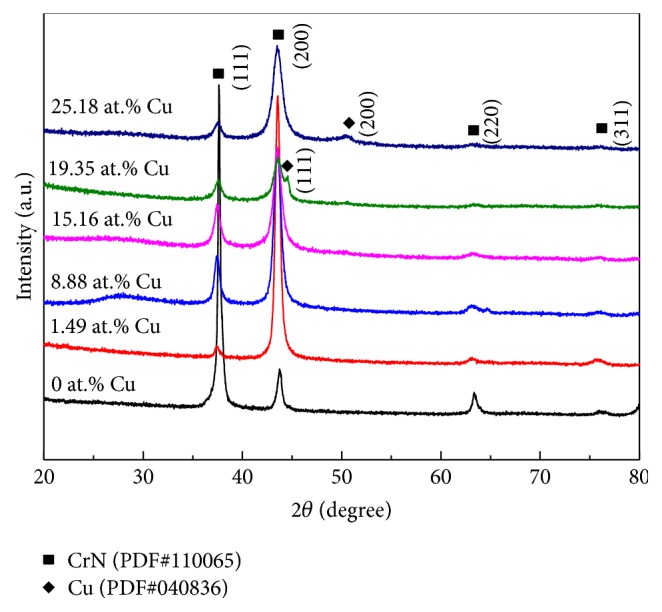
The XRD patterns of the Cr-Cu-N coatings as a function of Cu contents.

The XRD patterns of Cr-Cu-N coatings, deposited on Si wafer with various Cu target powers, are presented in Figure 3, with XRD spectrum of CrN coating being presented as reference. In the XRD spectrum of CrN coating, observed peaks according to the Joint Committee For Powder Diffraction Studies (JCPDS) card (PDF#110065) demonstrate a pattern of the FCC crystal structure with highest intensity of (111) plane. Other peaks such as (200), (220), and (311) also appear. As compared to the CrN coating, XRD patterns of the Cr-Cu-N coatings are mainly composed of CrN phase, and the (200) plane is the preferred orientation phase in Cr-Cu-N coatings. The (220) and (311) diffraction peak of the coating have disappeared when the Cu target power is up to 120 W, which demonstrates that Cu content has strong influence on coatings growth. The change of preferred orientation of the growth plane is known to be due to preferential sputtering of the atoms in different planes during the sputter deposition process. The (200) plane has the smallest nuclear stopping cross-section, which implies that it has the lowest sputtering yield in this plane. On the other hand, the (111) plane has the largest nuclear stopping cross-section; from sputtering theory it is known that the sputtering yield varies with nuclear stopping cross-section. On the first stage, the pure CrN coating would grow toward the orientation of the (111) plane with the lowest strain energy at high stress condition. Afterwards, increasing the sputtering power intensifies the preferential sputtering of the atoms in (111) planes, which suppress the growth of CrN grains along (111) plane [24]. Meanwhile, the (200) planes experience less sputtering which in turn promote the grains with a (200) orientation. With increase of Cu content, (111) and (200) diffraction peaks of CrN had the tendency to broaden. It was certified that Cu atoms addition could induce grain refinement, similar result to Zeman et al. [25] reported. However, there were no traces of metallic Cu and CuN phases that have been found in XRD diffraction patterns with Cu content below 15.16 (±0.23) at.%. It was considered that Cu atoms existed at the CrN grain boundaries as amorphous phase or Cu atoms incorporated in CrN crystals as solid solutions [26]; with Cu content further increasing, the crystalline (111) or (200) planes diffraction peaks of fcc-Cu were found, which was verified in PDF#040836.

**Figure 4:**
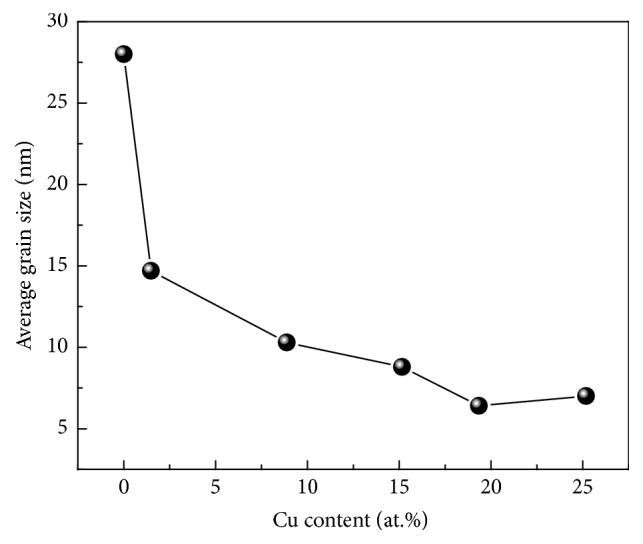
Average grain sizes of Cr-Cu-N coatings as a function of Cu contents.

**Figure 5:**
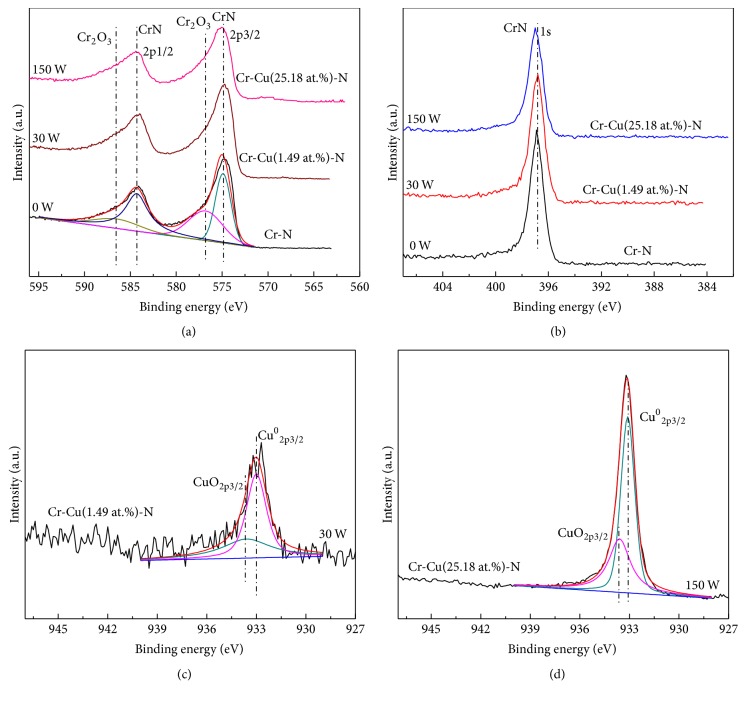
Binding energies of the Cr-Cu-N coatings with respect to the Cu contents (a) Cr 2p, (b) N 1s, and (c, d) Cu 2p.

Figure 4 shows the calculated average grain sizes of the Cr-Cu-N coatings by using Scherrer's formula [14]. The grain size of the coating deposited at CrN condition without Cu content has demonstrated maximum value of 28 nm and as the Cu content is further increased to 25.18 (±0.17) at.%, the grain size is decreased rapidly to ~7 nm. This result further indicates that an addition of Cu can block the grain growth, leading to grain size refinement effect, which is consistent with XRD result discussed above. It is well known that, with power increased, the coating obtains relative high energy by the ions bombardment, generating more defects. These surface defects increase the number of preferential nucleation sites which can also result in smaller grain sizes [27]. In order to confirm the Cu atoms existing form in the Cr-Cu-N coatings, the chemical bonding status was investigated by XPS. The binding energy (BE) of the C1s peak at 284.6 eV was considered as reference in calculating the binding energy.  Figure 5 shows the XPS spectra taken for Cr 2p, N 1s, and Cu 2p energy regions after an etched time of 180 s with various Cu contents. As shown in Figure 5(a), the Cr spectrum is fitted into four peaks: two peaks are at binding energies of 574.9 eV and 576.8 eV, which correspond to CrN and Cr_2_O_3_ of Cr 2p3/2 [28], while the other two peaks at 584.3 eV and 586.5 eV are for CrN and Cr_2_O_3_ of Cr 2p1/2 [29]. That means that the Cr atoms were reacted with N to form Cr-N bonds. No significant chemical shift has been detected with increasing Cu contents in the coatings. There is also a certain amount of Cr_2_O_3_ in the coatings, due to oxygen contamination coming from the sputtering chamber or target composition. In Figure 5(b), the N spectrum is found in the range of 396.8 ± 0.4 eV, which belongs to CrN phase [30]. The peaks found in the Cu 2p region are identified as metallic Cu (932.6 ± 0.4 eV) and oxide copper (934 ± 0.4 eV). Although a little peak shift exists, no distinct shoulder peaks appeared. It was reported that copper nitride synthesis needed considerable high energy, so it could be concluded that Cu existed as metallic species instead of copper nitride in the Cr-Cu-N coatings [31]. Because metal Cu is immiscible in the CrN phase, combined with XRD and XPS results, it can be concluded that the Cu as metallic species distributed in the interstitial of nanocrystalline CrN phase or segregated as amorphous phase in the intergranular boundaries with Cu content below 15.16 (±0.23) at.%, then Cu atoms grew up into metallic Cu crystallites agglomerated in the intergranular boundaries when Cu content was higher than 19.35 (±0.14) at.%. [31, 32].

**Figure 6:**
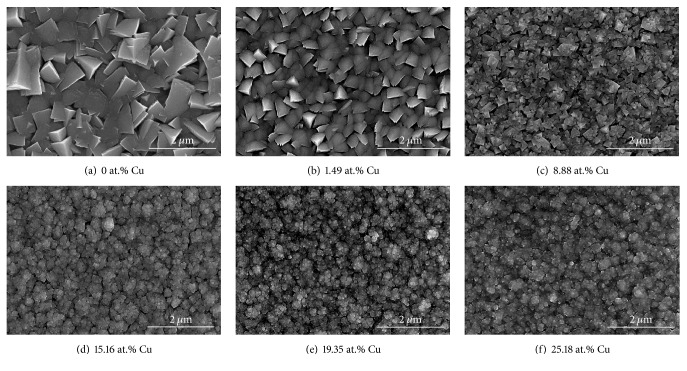
The surface morphologies of the Cr-Cu-N coatings with respect to the Cu content.

**Figure 7:**
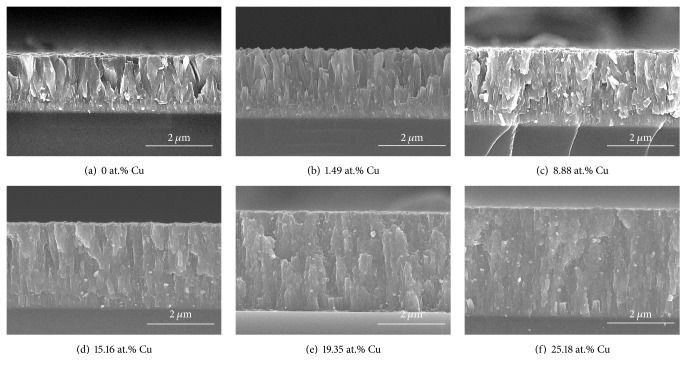
Cross-section images of the Cr-Cu-N coatings with various Cu contents.

To investigate the microstructures and thicknesses of Cr-Cu-N coatings as a function of Cu content, the surface and cross-sectional SEM images are shown as Figures 6(a)–6(f) and Figures 7(a)–7(f), respectively. As seen in Figure 6(a), the crystallites of the pristine CrN coatings are larger than others (Figures 7(b)–7(f)). When Cu atoms are incorporated into the CrN coatings, surface morphology is changed from irregular shape with triangular structure, which is typical columnar characteristic, to broccoli-like with compact structure, which is corresponding to variation of preferred orientation from CrN (111) to (200). It is clear that an addition of Cu content into the CrN coatings makes grain size finer. The thickness is gradually increased with the Cu target power from 0 to 150 W (see Figures 7(a)–7(f)). An obvious columnar structure is observed in Figures 7(a), 7(b), and 7(c), wherein small amount of Cu exists. When Cu content reached a relative high standard, the columnar structure becomes denser and the grain boundaries become vague, forming a nearly featureless structure. These phenomena can be explained as follows: with increase of the Cu target power, the surface adatom's energy and mobility were further increased, which forced them to move effectively due to ion bombardment compared with lower or no Cu target sputtering power; these movement particles would fill the voids between grains and interrupted the growth of large column structure; a higher deposition rate induces more Cu atoms, arriving at the surface of the substrate per unit time, and it is certainly producing more heterogeneous nucleation sites, resulting in the grain size refinement [27]. The Cu atoms are mobile on account of their immiscibility in CrN coatings and thus are able to disrupt the columnar grain growth during deposition process [17, 32], which can lead to controlling the size and shape of the grains.

**Figure 8:**
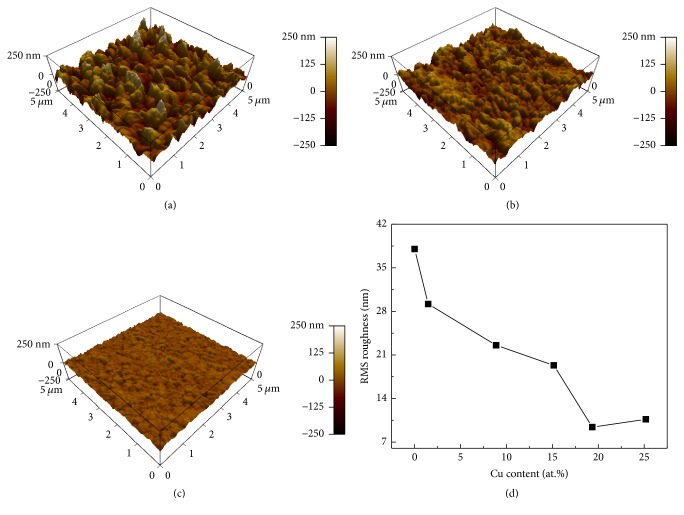
The 3D topographic AFM images (5 *μ*m × 5 *μ*m) of the coatings with Cu content of (a) 0 at.%, (b) 1.49 at.%, and (c) 25.18 at.%, respectively, and (d) plot of RMS roughness versus Cu content.

The typical AFM images (for three Cu powers) and surface roughness versus Cu content plot of Cr-Cu-N coatings are shown in Figures 8(a)–8(c) and 8(d), respectively. As it is expected from the images, when Cu content into coatings is increased, the surface roughness is rapidly decreased, which is in good agreement with the result found in Figures 6 and 7. The surface roughness value of coating is ~38 nm without Cu content; however, smoother surface can be obtained when the Cu is introduced. The smallest roughness value of <10 nm at 19.35 (±0.14) at.% of Cu is obtained which is related to an increase of an atomic movement and densification of the coatings as a result of high energy increased by target power.

### 3.3. Mechanical Properties

**Figure 9:**
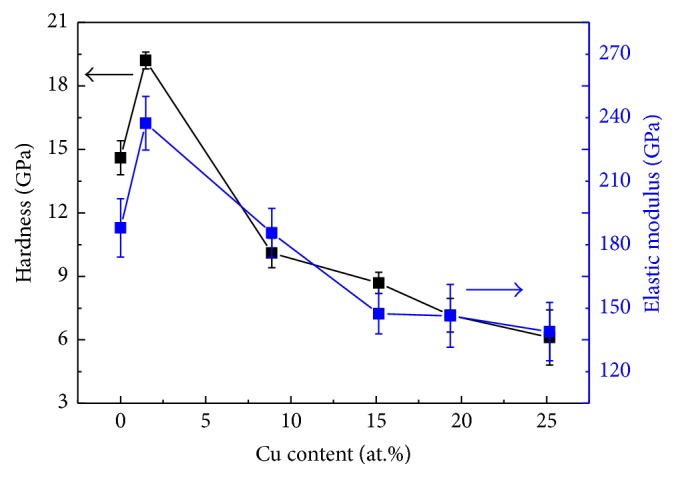
Variations of hardness and elastic modulus of Cr-Cu-N coatings as a function of Cu contents.

**Figure 10:**
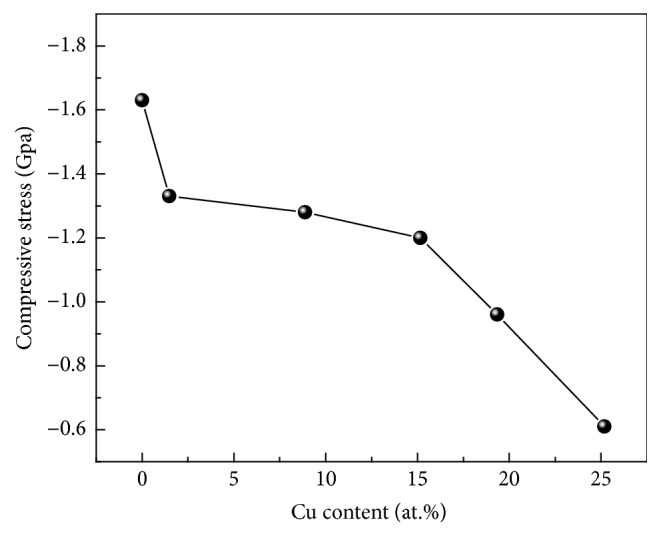
Compressive stress in the Cr-Cu-N coatings deposited with respect to the Cu content.

Average indentation hardness (*H*) and elastic modulus (*E*) of Cr-Cu-N coatings versus Cu contents are shown in Figure 9. The CrN coating exhibits nanohardness value of ~15 GPa and elastic modulus of ~188 GPa. Maximum hardness of ~20 GPa and elastic modulus of ~235 GPa are achieved when Cu content is 1.49 (±0.10) at.%. With increasing Cu content, the *H* value is decreased almost linearly from 20 GPa to 6 GPa. The same trend was also observed on the relationship between elastic modulus and Cu content of coatings. According to the Hall-Petch effect [33] applied in nanostructured materials, the hardness of materials increases with decreasing the grain size. The grain size calculation study done in Figure 4 has revealed that, with Cu addition, grain size refinement could be obtained. Therefore, both the solid solution strengthening (a few Cu atoms are dissolved in CrN crystals) [18] and Hall-Petch effect are responsible for the hardness enhancement. On increasing Cu content further, a great amount of softer metallic Cu phase formation takes place in the coatings and the grain size refinement reaches its critical value, which causes the inverse Hall-Petch effect. They were all contributed to the decrease of hardness. It is clearly seen that the Cu content plays a key role on the hardness and modulus of Cr-Cu-N coatings. In literature [15], Musil et al. reported that the hardness value of the Cr-Cu-N coating reached 35 GPa on adding 1 at.% Cu. On the research of Al-Cu-N nanocomposite coatings by Musil et al. [34], the maximum ~23 GPa H value at 5 at.% Cu has already been reported. Zeman et al. [25] revealed that the strong (111) preferred orientations with fine columnar structure and little Cu content are essential to achieve better hardness in Zr-Cu-N nanocomposite coating system. The increase in hardness of the coating with small addition of Cu was also observed in Mo_2_N coating system with adding 1–3 at.% Cu; with further increasing the Cu content, both the hardness and modulus dramatically declined [35]. These results reveal that by controlling the additive element content (i.e., Cu) one offers a convenient method to monitor the hardness and modulus of Me-Cu-N coatings.  Figure 10 shows the compressive residual stress date of the coatings decreased from 1.6 GPa at 0 at.% Cu to 0.6 GPa at 25.18 (±0.17) at.% Cu. It is suggested that the energetic ion bombardment by increasing Cu target power does not all the times generate an increased tendency of compressive stress during deposition process. This phenomenon can be explained by the addition of Cu, which otherwise can be from a buffer area, leading to generating stress relief in the coatings [36].

**Figure 11:**
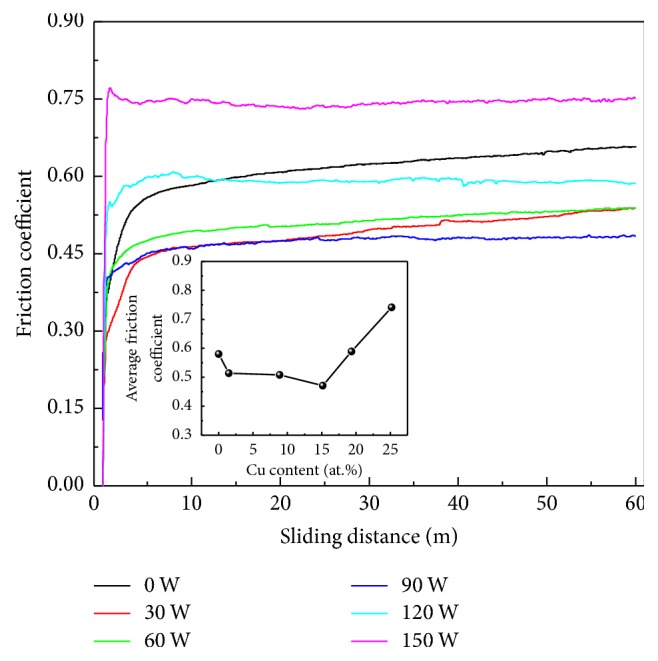
Friction coefficients of Cr-Cu-N coatings as a function of Cu content.

**Figure 12:**
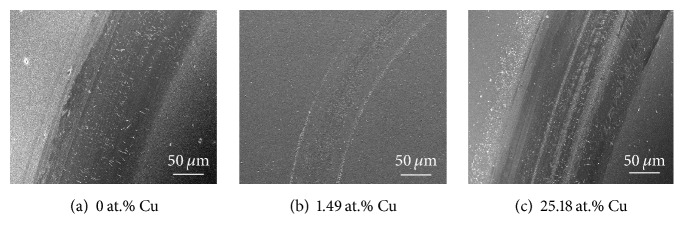
Typical worn morphologies of the Cr-Cu-N coatings with Cu content of (a) 0 at.%, (b) 1.49 at.%, and (c) 25.18 at.%.

Figure 11 shows the friction coefficients of Cr-Cu-N coatings with various Cu content against an Al_2_O_3_ ball on a ball-on-disk tribometer under a normal load of 2 N as a function of sliding distance at room temperature. The value of friction coefficient is increased sharply within the first 5 m and then stays constant in the following 55 m. The curves are not stable at the initial running-in stage and then become in the steady state. This behavior is in agreement with the basic wear principle where the effective value is obtained at the steady state position. Coating without Cu doping reveals relatively higher friction coefficient, that is, 0.58. With the Cu addition, the value of friction coefficient is decreased and the minimum friction coefficient of 0.47 is obtained for an addition of 15.16 (±0.23) at.% Cu content in the coating. The beneficial effect on tribology performance through adding Cu has previously been addressed by Shi et al. [37] by considering unidirectional scratch for a constant load. But in our work, when the Cu content exceeds a certain limitation, the friction coefficient rises rapidly as seen in inset image of Figure 11. That is because an excessive Cu causes softer phase coatings followed by poor hardness, which can be easily scuffed during rotate friction motion process. Otherwise, the separated chips (main is soft Cu phase) from substrate can be adhered to the grinding ball during the wear test, resulting in adhesive wear, which also can lead to increasing the value of the friction coefficient. Typical worn morphology of the CrN coating and Cr-Cu-N coatings with Cu content of 1.49 (±0.10) at.% and 25.18 (±0.17) at.% was in Figures 12(a)–12(c). Obvious furrows were found on the wear track, which were formed by transferring the peeled wear debris into the interface between the coating and friction ball in Figure 12(a); wear debris was also found and distributed on the wear track. The hard wear debris acted as the third body to be involved in the friction process and scratched the coating like a blade. Therefore, the main wear mechanism of CrN coating was abrasive wear. With doping 1.49 (±0.10) at.% Cu into the CrN coating as shown in Figure 12(b), the wear width and depth became more narrower and shallower. One reason for the slighter abrasion of the composite coating was its smaller friction coefficient and higher hardness. Also, it has been demonstrated that higher *H*/*E* ratio usually leads to better wear resistance [38], which is consistent with our result. When the Cu content reaches 25.18 (±0.17) at.% in the CrN coating as shown in Figure 12(c), the wear trace appeared more serious than that in Figures 12(a) and 12(b). That is the consequence of high friction coefficient, low hardness, and low *H*/*E* ratio. As mentioned above, excess softer Cu phase easily adhered to the grinding ball during the wear test resulting in adhesive wear.

**Figure 13:**
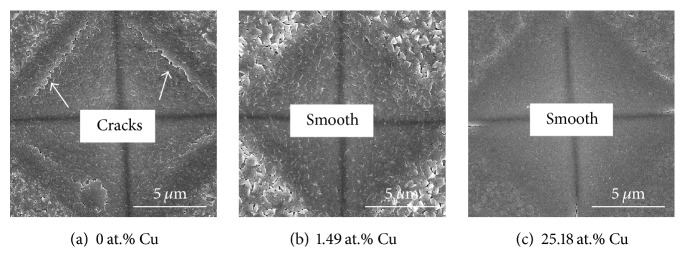
The crack resistances of Cr-Cu-N coatings with respect to Cu content.

Toughness is the ability of a material to absorb the energy during deformation up to its fracture. Many factors can influence the toughness of coating, including the doping content of third phase into coating; the residual stress of coating; and the ratio of hardness to elastic modulus of coating. In most cases, not just one factor plays a role on the toughness property, but two or several factors together contribute to the toughness. Using low content metal doping into the nitride coating is a common method for improving the toughness of coating, which is consistent with our result in Figure 13(b). It is cleared that the toughness of the containing low Cu content coating is better than that of the pure CrN coating (Figure 13(a)). The compact and dense structure makes a contribution to increasing the crack resistance of coating and that the excellent ductility of Cu may consume the energy from the compressive and shear stress by plastic deformation, which also can improve crack resistance [14]. When the Cu content is up to 25.18 (±0.17) at.%, the radial cracks were more serious than the pure CrN and 1.49 (±0.10) at.% Cu of CrN coatings, although the circumferential crack was absent. From [39], it is indicated that the value of *H*/*E* is an important criterion to evaluate radical crack; the more high value represents the high resistance against radial cracks. In this work, the ratio value of *H*/*E* was 0.07 with CrN and the maximum value 0.08 was obtained in 1.49 (±0.10) at.% Cu of CrN coating and then decreased to 0.04 when the Cu content is at 25.18 (±0.17)  at.% in CrN coating. From similar phenomena as reported in [39], we could conclude that excess metal Cu doping weakened the resistance of CrN coating to radial crack owing to low stress and low ratio between the hardness and elastic modulus of coating. Therefore, it appears that not all Cu contents are optimal. The underlying causes for this behavior need more investigation.

## 4. Conclusion

The effects of Cu contents on the microstructural evolution and properties of Cr-Cu-N coatings deposited by a RF/PDC comagnetron sputtering are studied and reported. The Cu content is varied from 0 to 25.18 (±0.17) at.%. The preferred orientations of these coatings are strongly affected by the Cu content; that is, pure CrN coating has demonstrated strong (111) plane, and with Cu addition, the (200) plane is dominating. When the Cu content reaches up to a certain value, the metallic Cu appears. That means that the Cu as metallic species distributed in the interstitial of nanocrystalline CrN phase or segregated as amorphous phase in the intergranular boundaries with low Cu doping content, while it precipitated as metallic Cu phase and agglomerated in the intergranular boundaries at high Cu doping content. The friction coefficient of the coatings was decreased by Cu addition. The hardness and elastic modulus values decreased from 20 GPa and 235 GPa to 6.1 GPa and 142 GPa, respectively, as the Cu content increased from 1.49 (±0.10) to 25.18 (±0.17) at.%. The CrN coating with 1.49 (±0.10) at.% Cu addition presents excellent tribological properties, which would be attributed to the lower friction coefficient, high hardness, and high *H*/*E* ratio. Proper Cu addition, improved hardness, and enhanced toughness can be achieved at the same time.

## Figures and Tables

**Table 1:** The deposition parameters and the thickness of the coatings.

Deposition parameters						
Base pressure (Pa)	6.0 × 10^−3^					
Working pressure (Pa)	0.4					
N_2_ gas flow (sccm)	20					
Ar gas flow (sccm)	65~80					
Substrate temperature (°C)	300					
Substrate bias voltage (v)	−150					
Deposition time (min)	120					
Cr target power (w)	800	800	800	800	800	800
Cu target power (w)	0	30	60	90	120	150
Coating thickness (*μ*m)	1.71	2.16	2.30	2.52	2.92	3.22
